# Supplementary material for: Development and Initial Testing of an Artificial Intelligence-Based Virtual Reality Companion for People Living with Dementia in Long-Term Care
Source: J Clin Med. 2024 Sep 20;13(18):5574. doi: 10.3390/jcm13185574 (PMC11432461; doi:10.3390/jcm13185574)
Supplement: Supplementary file 1 [file jcm-13-05574-s001.zip › S1 Resident Participant Questionnaire v2.pdf]

Virtual Reality Artificial Companion for Persons with Dementia
[questions for person with dementia (resident participant])

Thank you for taking the time to answer some questions about your experience with the virtual reality companion.

(For each question, let the person respond, then probe further…)

1. Did you like using the goggles? Why? Why not? Was it easy to use? Was it comfortable? How can we make the goggles better?

2. Did you like the companion? Did you like talking to him/her? Why? Why not?

3. Do you think that talking with the companion improved your day? Why? Why not? How can we make the companion better?

4. Would you like to use the goggles again? Why? Why not?
