# Supplementary material for: Development and Initial Testing of an Artificial Intelligence-Based Virtual Reality Companion for People Living with Dementia in Long-Term Care
Source: J Clin Med. 2024 Sep 20;13(18):5574. doi: 10.3390/jcm13185574 (PMC11432461; doi:10.3390/jcm13185574)
Supplement: Supplementary file 1 [file jcm-13-05574-s001.zip › S2 Staff Participant Questionnaire.pdf]

Virtual Reality Artificial Companion for Persons with Dementia
[questions for staff participant]

Thank you for taking the time to answer some questions about your experience with the virtual reality companion.

(For each question, let the person respond, then probe further…)

1. What has been your experience helping or watching the resident use the virtual reality system (i.e. the physical VR goggles)?
   What aspects of the experience were positive?
   What aspects of the experience were negative?

   Was it easy to use?
   Was the resident able to wear the headset comfortably?

2. What has been your experience watching the resident interact with the virtual companion (avatar)?
   What aspects of the experience were positive?
   What aspects of the experience were negative?

   Did the resident interact with the VR companion?
   Did the resident speak to the avatar?

3. Do you think that interacting with the virtual companion improved the resident's day?
   Can you give some examples of how?

4. Do you have any suggestions for how we could improve the use of the virtual reality goggles and the virtual companion for people living in long-term care?

5. Do you think that it would be good for the resident to continue to use the virtual reality goggles and the virtual companion after this study is over?
   What would help them to continue to use it?
   What would be a challenge if they wanted to continue using it?
